# Identifying brain-penetrant small-molecule modulators of human microglia using a cellular model of synaptic pruning

**DOI:** 10.1038/s41386-025-02123-1

**Published:** 2025-05-09

**Authors:** Liam T. McCrea, Rebecca E. Batorsky, Joshua J. Bowen, Hana Yeh, Jessica M. Thanos, Ting Fu, Roy H. Perlis, Steven D. Sheridan

**Affiliations:** 1 Center for Genomic Medicine and Department of Psychiatry, Massachusetts General Hospital, Boston, MA USA; 2 Tufts Institute for Artificial Intelligence, Tufts University, Medford, MA USA; 3 Department of Psychiatry, Harvard Medical School, Boston, MA USA

**Keywords:** Neuroscience, Developmental biology, Psychiatric disorders

## Abstract

Microglia dysregulation is implicated across a range of neurodevelopmental and neurodegenerative disorders, making their modulation a promising therapeutic target. Using PBMC-derived induced microglia-like cells (piMGLCs) in a scalable assay, we screened 489 CNS-penetrant compounds for modulation of microglial phagocytosis of human synaptosomes in a validated assay for microglia-mediated synaptic pruning. Compounds from the library that reduced phagocytosis by ≥2 standard deviations across the library without cytotoxicity were validated in secondary screens, with 28 of them further confirmed to reduce phagocytosis by 50% or more. These compounds comprise a wide range of therapeutic classes with different mechanisms of action, including immunosuppressants, kinase inhibitors, antipsychotics, and epigenetic modulators. Image-based morphological measurements were calculated to measure the degree of ramified vs. ameboid morphotypes as an indicator of activation state. Additionally, transcriptomic profiling indicated divergent effects on cell signaling, metabolism, activation, and actin dynamics across confirmed compounds. In particular, multiple CNS-penetrant small molecules with prior FDA approval or demonstration of safety in vivo demonstrated modulatory effects on microglia. For example, identified drugs such as the tyrosine kinase inhibitors lapatinib, alectinib, and lazertinib and the epigenetic modulator vorinostat have been approved for various cancer treatments and are being investigated for other indications; however, they have not been extensively studied in patients for neurodevelopmental and neurodegenerative disorders. These potential disease-modifying agents represent high-priority candidates for repositioning studies in neurodevelopmental, neuroinflammatory, or neurodegenerative disorders.

## Introduction

Microglia have essential functions in the developing and mature central nervous system (CNS) [1, 2]. After populating the brain during early embryonic development [1, 3], they play a central role in immunosurveillance [4, 5], regulation of neuronal progenitor populations [6], neurogenesis [7, 8], and shaping synaptic networks via selective pruning [9–11].

Convergent lines of evidence, including animal studies, post-mortem and in vitro human models, associate dysregulated microglia-mediated synaptic pruning with the pathophysiology of CNS disorders [12–14]. In particular, recent investigations have shown that dysregulated microglial phagocytosis, such as during synaptic pruning, may be associated with neurodevelopmental disorders including bipolar disorder, autism, and schizophrenia [12, 15, 16]. Post-mortem studies demonstrate reduced cortical dendritic spine density within the brains of individuals with schizophrenia [15–17], consistent with clinical structural neuroimaging studies [18, 19]. Parallel lines of evidence from rodent models and human genomics suggest that these disorders may arise, in part, from dysfunctional microglia-mediated pruning [14, 20]. Microglial processes may amplify or exacerbate the downstream effects of these disorders even when such disease states are initiated by other processes [21].

For example, microglia become activated upon initial accumulation of amyloid-beta (Aβ) plaques in Alzheimer’s Disease (AD), leading to an increased release of pro-inflammatory cytokines, reactive oxygen species (ROS), and other neurotoxic substances [22]. This chronic neuroinflammatory response exacerbates neuronal damage and accelerates disease progression [23]. Conversely, in a mouse AD model, inhibition of phagocytic microglia has been shown to reduce the extent of early synapse loss [24].

Interventions that modulate microglial function are therefore promising candidates for disease-modifying treatments. While nonspecific anti-inflammatory agents have been investigated clinically with varied efficacy [22, 25, 26], few small-molecule microglia modulators have been specifically identified, and even fewer have been investigated mechanistically.

In vitro assays utilizing human microglia represent a promising means for the discovery of new compounds or the repurposing of existing therapeutics that modulate microglial function. However, due to their limited availability, microglia derived from post-mortem or surgical biopsy brain material lack the scalability to perform high-throughput screening (HTS). These limitations have led to the development of alternative microglia-like cellular models, such as from the differentiation of human induced pluripotent stem cells (iPSCs) [27]. These iPSC-derived microglia models, however, also have limitations, including the requirement for epigenetic resetting to accomplish pluripotency, resulting in more fetal-like cell states upon differentiation, extensive optimization required for large-scale in vitro derivation, expensive media over extended time in culture, and significant technical artifacts after scaling due to high line-to-line and batch-to-batch variability. Another strategy utilizes the direct reprogramming of adult human peripheral mononuclear blood cells (PBMCs) to microglia-like cells (piMGLCs) [28, 29] and reviewed in [27]. While direct reprogramming does not recapitulate microglial developmental ontogeny as with iPSC-derived models, the monocyte fraction of PBMCs possesses high phenotypic plasticity and can adopt a robust microglial-like phenotype in vitro. This direct conversion of human monocytes to induced microglia-like cells (piMGLCs), therefore, represents a promising strategy for generating large-scale assays amenable to HTS from human blood [14, 28–30].

We scaled our high-content, image-based assay for functional synaptic pruning utilizing the engulfment of human neural culture-purified synaptic vesicles (synaptosomes) by piMGLCs [14, 30, 31] to screen a library of known CNS-penetrant compounds. To investigate mechanisms of action, we secondarily characterized morphology as well as altered transcriptomic pathways.

As many of these compounds have previously been investigated in human studies for other indications, with some already in clinical use, we aimed to identify high-priority molecules with known CNS penetrance and safety for repositioning in psychiatric and neurodevelopmental disorders.

## Materials and methods

### Ethical statement

The study was approved by the Mass General Brigham Institutional Review Board (IRB). Informed consent was obtained from all participants, and samples have been deidentified.

### PBMC-derived induced microglia-like cell (piMGLC) culture

PBMCs were plated at 400,000 cells/cm² in tissue culture-treated 6-well plates (Corning, #353046) pre-coated with Geltrex (Gibco, #A1413202) in RPMI-1640 medium (Sigma #R8758) supplemented with 10% heat-inactivated FBS (Sigma #12306C) and 1% Pen/Strep (Life Technologies #15140-122). After 24 h, the media were replaced with RPMI-1640 containing 1% Pen/Strep, 1% Glutamax (Life Technologies #35050-061), 100 ng/mL IL-34 (Biolegend #577904), and 10 ng/mL GM-CSF (PeproTech #300-03). After 10 days, conditioned culture media were collected and retained. (piMGLCs) were harvested using Accutase (Sigma, #A6964). Cells were resuspended in retained media at 50,000 cells/ml and plated into 96-well tissue culture plates (Corning, #3904) at a density of 30,000 cells/cm^2^ (10,000 cells per well in 200 µl) for 4 days before assaying.

### Compound treatments and phagocytosis assays

piMGLCs were pretreated for 24 h with a CNS-penetrant compound library (MedChemExpress #HY-LO28) (Supplementary Data Table S1) at a final concentration of 10 μM, with some exceptions at 2 μM (Supplementary Data Table S2). For the primary screen, three replicate treatments of each compound were split across different plates. The secondary screen included piMGLC plates treated with 47 compounds for 24 h (Supplementary Data Table S4).

Phagocytosis assays were performed with purified human synaptosomes labeled with pHrodo-Red dye (Invitrogen #P36600), washed, sonicated, and added to wells at a final concentration of 3 µg/well. After 3 h, assays were concluded by fixing cells with 4% paraformaldehyde (Electron Microscopy Sciences, #15713S).

### High-content image analysis

Confocal images were analyzed with CellProfiler (Version 4.2.1) [32]. Images were corrected for background signal, and nuclei, cytoplasm, and synaptosomes were segmented. Data were filtered to exclude segmentation errors or toxicity artifacts. The phagocytic index was calculated as synaptosome area divided by cell count. Data were cleaned in R, applying filters to exclude images with over-segmentation or treatment-induced toxicity.

### Multiplexed RNA-seq and data analysis

96-well multiplexed libraries (DRUG-seq [33]) were prepared using the MERCURIUS^™^ DRUG-seq kit (Alithea Genomics, #10841) following the manufacturer's instructions. Cells were lysed, and RNA was reverse-transcribed using well-positioned, specific barcoded oligo-dT primers (Alithea Genomics, 10513). First and second-strand cDNA were synthesized, purified, and loaded onto a NovaSeq X Plus 1.5B read flow cell and sequenced on the NovaSeq X Plus instrument.

Sequencing data were aligned to the reference genome hg38 using STAR v. 2.7.11b [34] using “solo” mode. Differential expression analysis was performed using a modified version of the DRUG-seq analysis pipeline [35] and DESeq2 [36] to perform differential expression analysis. Functional enrichment analysis was conducted using QIAGEN Ingenuity Pathway Analysis v. 24.0.1 [37] canonical pathway analysis.

More detailed methods are available in Supplementary Material and Methods.

## Results

### PBMC-derived induced microglia-like cells as a scalable HTS platform for identifying modulators of synaptosome phagocytosis

**Fig. 1:**
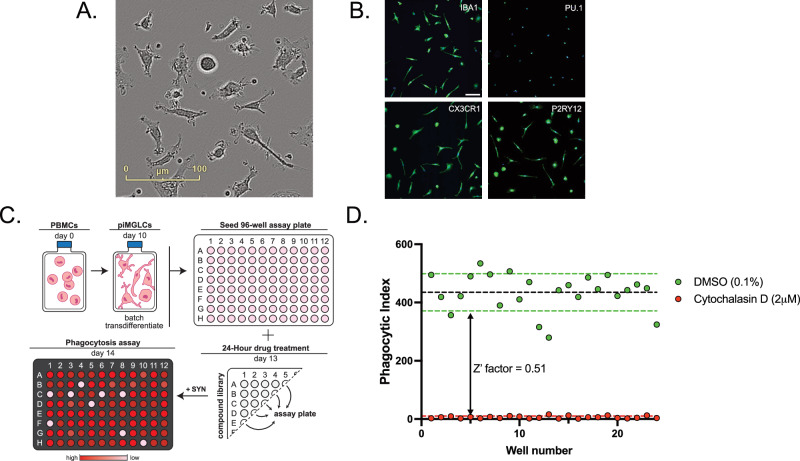
Derivation of PBMC microglia-like cells (piMGLCs). **A** Representative phase-contrast image of piMGLCs. Scale bar, 100 μm. **B** Confocal images of piMGLCs stained for indicated canonical microglial markers IBA1, PU.1, CX3CR1, and P2RY12. Scale bar, 100 μm. Image-based phagocytosis assay optimization for CNS-penetrant compound HTS screening. **C** Overview workflow for large-scale batch derivation of piMGLCs and 96-well functional synaptosome phagocytosis image-based screens. **D** Z’ factor = 0.51 was determined using 0.1% DMSO (green) as negative control and 2 μM cytochalasin D (red) as positive control phagocytosis inhibitor, 24 wells of each. Data points represent phagocytic indices of each well, calculated as the sum of synaptosome area divided by the sum of cell count within 12 image fields per well. Black dashed lines are the group means, and colored dashed lines signify one standard deviation around the mean for the group of the corresponding color.

We have previously optimized and validated a cytokine induction method to derive PBMCs into piMGLCs [14, 30]. For large-scale derivation of piMGLCs used in our assays, we generated a biobank of assay-ready cells in a single large batch of PBMCs (approx. 3 × 10^9^ cells) from a leukapheresis preparation to minimize batch effects in the screening assays. piMGLC batches were induced for ten days before being harvested, pooled, and replated into 96-well assay plates. After three days, the majority of piMGLCs displayed a ramified morphology (Fig. 1A), with positive immunostaining for the microglial markers IBA1, PU.1, CX3CR1 and P2RY12 (Fig. 1B). We have previously demonstrated that these piMGLCs demonstrate a high degree of transcriptomic similarity to primary microglia, immortalized primary microglia, and isolated post-mortem microglia [30].

To screen for potential modulators of microglia-mediated synaptic pruning, we quantified phagocytosis of synaptosomes isolated from large-scale human iPSC-derived differentiated neuronal cultures and labeled with a pH-sensitive dye (pHrodo) that fluoresces red in the acidic post-phagocytic phagolysosome compartments. The lack of fluorescence outside the cell enables robust quantification of cellular uptake. We used these reagents in conjunction with the piMGLCs in an in vitro model of microglial synaptic engulfment (Fig. 1C). After incubation with synaptosomes for 3 h, cells were fixed and stained with the microglial marker IBA1 to identify cellular perimeters. This cell segmentation, along with the quantification of red fluorescence within cells, indicating uptake of pHrodo-labeled synaptosomes [14, 30], was performed using confocal microscopy images input into an automated image analysis pipeline in CellProfiler [32].

To determine assay robustness in 96-well plate format, we treated 24 wells each for 24 h with either only 0.1% DMSO (negative carrier control) or 2 µM cytochalasin D (CytoD), a cell-permeable inhibitor of actin polymerization, used as a positive control treatment to inhibit phagocytosis. Phagocytosis activity (expressed as phagocytic index, see “Materials and methods” section) in positive control and negative untreated wells was determined to have a robust Z-factor score of 0.51 (Fig. 1D), indicating a high-quality assay for screening [38]. For further statistical confirmation of assay robustness, we determined the Strictly Standardized Mean Difference (SSMD) = 9.43, above the accepted value of >5, indicating a highly robust assay [39, 40].

### Functional CNS-penetrant compound screening identifies compounds that modulate synaptosome phagocytosis

We performed image-based primary compound screening, allowing for the screening of compounds that affect microglia function as indicated by phagocytosis of isolated synaptosomes in a model of synaptic pruning [14, 30]. Using our optimized assay in 96-well plate format, we screened a CNS-penetrant small-molecule library of 489 compounds (Supplementary Data Table S1) by treating for 24 h before the addition of labeled synaptosomes to initiate the assay.

**Fig. 2:**
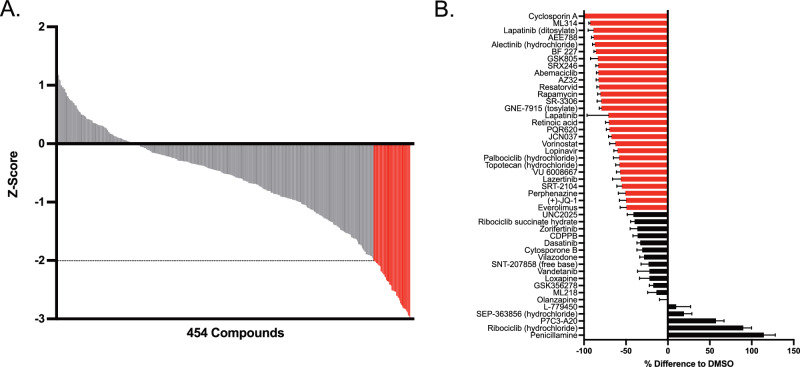
Image-based compound screen for synaptosome phagocytosis modulators in piMGLCs. **A** Primary screen of 489 CNS-penetrant compounds assayed at 10 μM resulted in 454 measurable phagocytic activity (35 removed due to toxicity). 47 compounds that were inhibited by a Z-score compared to DMSO (Z-score = 0) of −2 or below are indicated in red. **B** Secondary confirmation screen of hit compounds measured as the ratio of mean phagocytic index per image field (*n* = 15) of indicated compound treatment to mean DMSO only control. Compounds with a ratio of 0.5 or less are indicated in red. Error bars indicate SEM.

Treated wells containing below a threshold number of remaining cells (<10% compared to DMSO) were considered to indicate possible toxicity (Supplementary Data Table S3). After excluding 35 compounds (7%) with this initial filter, 454 compounds remained for further phagocytic activity determination after eliminating these wells. We further prioritized compounds more stringently based on those that deviated from the mean phagocytic index of the DMSO negative control by at least a Z-score of ±2, or two standard deviations (2SD), as initial primary hits. We did not identify any activators with a Z-score ≥2 using this method of prioritizing hits, but were able to identify 47 compounds (~10% of the entire library) that stringently (Z-score ≤ −2) inhibited phagocytosis of synaptosomes (Fig. 2A and Supplementary Data Table S3).

To confirm results from the primary library screen and demonstrate independent reproducibility, we performed a secondary confirmation screen on the 47 hit compounds with freshly prepared bulk compound stocks. One compound, MK-28, was determined to be toxic, and six compounds were shown to either increase phagocytosis (penicillamine, ribociclib HCl, P7C3-A20, SEP-363856 and L-779450) or have no measurable effect (olanzapine) when compared to carrier (DMSO) negative control measures (Fig. 2B). While the remaining 40 compounds all reduced synaptosome phagocytosis as was observed in the primary screen, 28 compounds demonstrated a 50% reduction or greater when compared to the DMSO control confirming these compounds as inhibitors with apparent IC50 values of ≤10 μM (Supplementary Data Table S4). The comparison of the measured phagocytic indices for the forty remaining inhibiting compounds between the primary and secondary screening results revealed a moderate correlation for most compounds (*R* = 0.3, *p* = 0.067 across compounds, Supplementary Fig. S1), with some outliers reflecting possible subtle differences between drug concentrations in initial primary screen compound library versus independent individual secondary screen compounds.

### Phenotypic compound screening identifies CNS-penetrant compounds that modulate microglial morphology

In the adult brain, microglia typically exist in a surveillant state characterized by a ramified morphology [4]. Microglia undergo morphological changes into a more ameboid shape upon activation due to various responses and disease states. This transformation can be used as a proxy for activation state [41], with more ramified or ameboid morphologies representing a surveillant or more activated state, respectively.

**Fig. 3:**
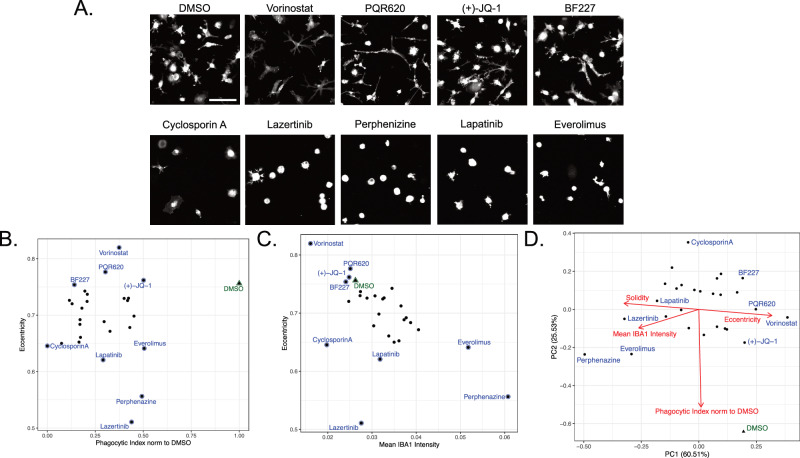
Phenotypic screening identifies compounds that modulate piMGLC morphology. **A** Representative images of DMSO vehicle control and select compound treatments resulting in ramified (top row) or ameboid (bottom row) morphologies. **B** Phagocytic index versus eccentricity for compounds ≤50% DMSO control phagocytic index in secondary screen. **C** IBA1 intensity versus eccentricity for compounds ≤50% DMSO control phagocytic index in secondary screen. Each dot in (**B**) and (**C**) is the mean value of 5–15 image fields with indicated compounds corresponding to images in (**A**) for representation. **D** Principal Component Analysis (PCA) of functional and morphological features over compounds exhibiting ≤50% DMSO control phagocytic index. PC loading vectors in red demonstrate the relationship between features.

An advantage of our image-based arrayed screen is its versatility, as it allows for the determination of multiple parameters of the cellular response in parallel to phagocytosis upon compound treatment. Using CellProfiler [32], we examined cellular morphologic parameters in the IBA1+ stained cells to additionally determine measures of solidity and eccentricity (see Supplementary Methods) in the compound-screened piMGLCs (Fig. 3). We have previously shown that these morphometric parameters allow for the high-throughput categorization of individual cells into ramified (low solidity, high eccentricity), ameboid (high solidity, low eccentricity) and in-between bipolar, or rod-shaped (mid solidity, mid eccentricity) morphotypes [31].

Compounds that reduced phagocytosis by 50% or more in the secondary confirmation screen resulted in varied responses in cell morphology (Fig. 3A and Supplementary Data Table 4). For example, though they share low phagocytic indices, vorinostat treatment increased cell ramification as shown by increased eccentricity (Fig. 3B and Supplementary Fig. S2A) and reduced solidity (Supplementary Figs. S2B and S3A) compared to the DMSO negative control, whereas perphenazine treatment conversely resulted in exclusively compact ameboid morphology. Cyclosporin A, the compound resulting in the greatest phagocytic inhibition upon treatment, also decreased eccentricity, though not to the same extent. Taken together, these observations suggest the mechanisms by which these compounds modulate phagocytosis of synaptosomes have divergent effects on microglia morphology and, by proxy, activation state.

We also examined IBA1 intensity as a potential indicator of activation state. While IBA1 is a widely used marker to identify microglia, it is not exclusively tied to activation as it is constitutively expressed in these cells under both resting and activated conditions. However, increased IBA1 levels may indicate more activated microglia when taken in conjunction with other parameters such as phagocytic activity and morphology [42, 43] or additional activation markers [44]. To this end, we further analyzed the correlation between these parameters in our image-based secondary screen. With a few exceptions, such as lazertinib and cyclosporin A, we observed that compound treatments resulting in higher eccentricity morphology corresponded with lower IBA1 intensity (*R* = −0.565, *p* = 0.0014) (Fig. 3C). The opposing trend was observed using the related parameter of solidity (*R* = 0.624, *p* = 0.0003) (Supplementary Fig. S3B). The relationship between multiple morphological and phenotypic variables can be summarized using Principal Component Analysis (Fig. 3D). As expected, solidity and eccentricity are highly negatively correlated, as indicated by parallel and opposite principal component loading vectors. Conversely, the Phagocytic Index loading vector appears as nearly perpendicular to the solidity-eccentricity axis, indicating that it is uncorrelated. Mean IBA1 intensity correlates most strongly with solidity, as shown in Fig. 3D.

Taken together, phenotypic characterization of the 28 compounds prioritized in our secondary confirmation screening shows differential effects on morphology as a proxy for cell state, suggesting different mechanisms resulting in decreased phagocytic activity.

### Transcriptomics identifies divergent pathways affecting phagocytosis and activation state

**Fig. 4:**
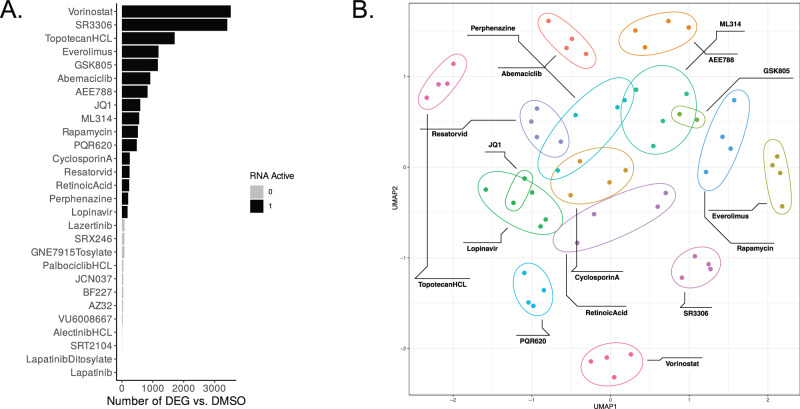
Drug-Seq transcriptomic results for secondary screen. **A** Number of differentially expressed genes (DEG) resulting from the comparison of all replicates of a given compound vs. select DMSO replicates (see Supplementary Methods). Active RNA compounds had more DEG than 95% of the DMSO vs. DMSO comparisons. **B** UMAP of active RNA compounds shows grouping of replicates.

**Fig. 5:**
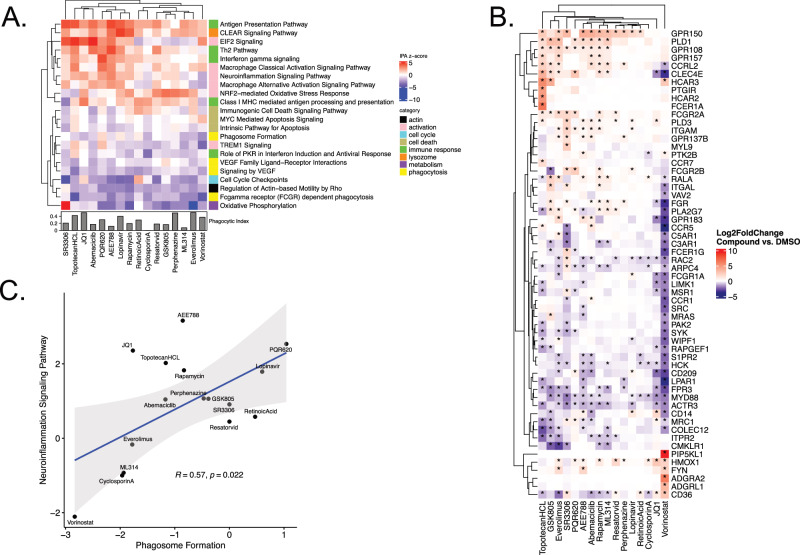
Transcriptomic pathway analysis demonstrates the diversity of compound treatment responses. **A** Activation/Suppression z-score for select IPA Canonical Pathways that are enriched in the DEG of Drug-Seq compounds. **B** Heatmap of gene differential expression in the Phagosome Formation IPA Pathway by compound. Due to the large number of differentially expressed genes (DEG) in this pathway, only the top DEG are shown (adjusted *p*-value < 1e^−6^ and absolute log_2_ fold-change relative to DMSO >2). Heatmap color represents gene expression level (log_2_ fold-change relative to DMSO), *indicates a significant DEG with adjusted *p*-value < 0.1 and absolute log2 fold-change >1. **C** Relationship between phagosome formation and neuroinflammation pathways for RNA-active compounds exhibiting ≤50% DMSO control phagocytic index, showing the unique position of vorinostat.

To investigate the potential mechanism of action of the 28 compounds confirmed in the secondary screen, we performed DRUG-seq [33], a high-throughput multiplexed next-generation RNA sequencing method to measure the effect of 24-h compound treatment on cellular transcriptional programs in piMGLCs. In all, 16/28 compounds were determined to have a measurable effect on transcriptional activity (“RNA-active”, see Supplementary Methods). These 16 “RNA-active” compounds were prioritized for further analyses (Fig. 4A), with the highest number of differentially expressed genes (DEG) observed in vorinostat-treated wells (3525). We visualized the grouping of replicate wells using Uniform Manifold Approximation and Projection (UMAP), which demonstrates reproducibility of the treatment effects across wells and batches, with tighter grouping of replicates observed for compounds which had a stronger effect on transcription (Fig. 4B, Supplementary Fig. S4). Functional enrichment analysis performed with QIAGEN Ingenuity Pathway Analysis (IPA) shows diverse effects of the compounds (select categories shown in Fig. 5A), full list given in Supplementary Data Table S6.

All compounds suppressed gene pathways involved in Fc gamma-receptor-dependent phagocytosis, most strongly vorinostat, and most compounds suppressed gene pathways associated with phagosome formation, with the exception of SR3306, PQR620, lopinavir, topotecan HCL, and retinoic acid. A closer examination of differentially expressed genes in the phagosome formation pathway provides insights into the distinct mechanisms by which the compounds reduce phagocytosis (Fig. 5B shows the most significantly impacted genes in this pathway). Genes involved in the regulation of actin cytoskeleton were most uniformly downregulated, including actin-related proteins *ACTR3* and *ACTR2* (not shown), as was MYD88 and FPR3, two other key genes in phagosome formation, regulating immune signaling and migration. FCGR genes were also differentially impacted across compounds. *FGR* was upregulated in 6/16 and down in 2/16, including vorinostat. *FCGR1A* was downregulated in 3/16 and upregulated in only lopinavir. *FCG2RA* was upregulated in 7/16, most strongly in everolimus, SR3306, and PQR620. Of note, phagocytosis-related genes Cytoplasmic FMR1 interacting protein (CYFIP) family genes *CYFIP1* and *CYFIP2*, involved in actin polymerization, were also impacted, but in divergent ways across compounds. *CYFIP1*, which we recently demonstrated to be involved in microglial phagocytosis, morphology, and motility [31], was downregulated in 3/16, including vorinostat, which strongly upregulated *CYFIP2*.

Regulation of actin-based mobility by Rho was also suppressed by all compounds except SR3306, and the pattern of suppression closely mirrored the suppression of phagocytosis. This is consistent with the established role of actin remodeling in phagocytosis [45]. VEGF signaling pathways were similarly broadly suppressed across compounds. In contrast, cell activation pathways featured prominently among activated pathways, including Classical and Alternative Macrophage Activation, Neuroinflammation, EIF2 Signaling, NRF2-mediated Oxidative Stress Response, and TREM1 Signaling. The degree of activation varied among compounds, with the strongest overall activation observed for SR3306, topotecan HCL, JQ1, abemaciclib, PQR620, AEE788, and lopinavir. In a less activating group of compounds, including cyclosporin A, resatorvid, GSK805, perphenazine, everolimus, and ML314, EIF2 signaling was decreased, while NRF2-mediated oxidative stress response was increased. This pattern suggests a potentially balancing interaction between the autophagy and oxidative stress responses [46].

Antigen presentation pathways were activated to varying extents across compounds, except for suppression of Class I MHC-mediated antigen processing and presentation in SR3306. SR3306 overall showed the most distinct response from the group of RNA-active compounds, with strongly activated Oxidative Phosphorylation and strongly suppressed Cell-cycle checkpoint pathways.

A highly promising candidate therapeutic is one that could maximize suppression of phagocytosis without causing high levels of cell activation, minimizing the risk of off-target effects on microglia. IPA z-scores for phagosome formation-related and neuroinflammation-related gene expression displayed a moderate correlation across compounds (Fig. 5C), and vorinostat was identified as one such compound that strongly suppressed phagosome formation without increasing neuroinflammation gene pathways.

## Discussion

In this functional compound screening of a CNS-penetrant compound library, we initially identified 47 compounds that modulate microglia-mediated phagocytosis of synaptosomes in an in vitro model of synaptic pruning [14, 27, 30], of which 28 demonstrated consistent inhibitory effects in secondary confirmation screening. Notably, while these confirmed hit compounds all inhibit phagocytosis in microglia-like cells, they exhibit variability in additional phenotypic screening, including differential effects on cellular morphology and transcriptomic effects, indicating multiple mechanisms of action rather than reflecting a single common pathway. These compounds cover a wide range of therapeutic classes, each with different mechanisms of action: immunosuppressants, kinase inhibitors, neurotransmitter modulators, including antipsychotics, and epigenetic modulators. Despite these mechanistic differences, all the compounds diminish microglial phagocytosis, suggesting the potential to reverse microglia-mediated pruning dysregulation implicated in multiple neurological disorders [47, 48]. While this in vitro assay models microglia-mediated synaptic pruning amendable to high-throughput screening, a limitation is that it lacks complex multicellular interactions, warranting further follow-up in neuronal co-cultures or organoids. Additionally, compound concentrations used in this in vitro HTS screen do not reflect the most efficacious physiological concentrations, and cellular specificity would have to be optimized in vivo.

Many of the compounds confirmed to reduce microglial phagocytosis in our screen are current FDA-approved medications [14/28; 50%] or have been investigated in human studies [4/28, 14%] (Supplementary Data Table S5). Immunosuppressant compounds that show robust microglia phagocytosis reduction, such as cyclosporin A and rapamycin, are effective in preventing organ rejection in transplant recipients [49]. Several identified kinase inhibitors in the screen are currently used for cancer treatment (e.g., lapatinib, alectinib, abemaciclib, and recently FDA-approved lazertinib) either alone or in combination with other therapeutics. Perphenazine, loxapine, and vilazodone have been indicated for psychiatric diagnoses, including schizophrenia, bipolar disorder, and major depression. While their primary mechanism of action is best understood for their effects on neurotransmission, substantially less is understood about the indirect effects of these compounds on microglial function. Conversely, 10/28 (36%) of confirmed hit compounds are investigational or experimental without any current clinical application. These include such the c-Jun N-terminal kinases (JNK) inhibitor SR3306, investigated for dopaminergic neuron protection in animal models of Parkinson’s disease (PD) [50] and the leucine-rich repeat kinase 2 (LRRK2) inhibitor GNE-7915, which along with its derivatives showed promising pharmacokinetic and safety profiles in rodent and non-human primate models for potential application of LRRK2 inhibition in PD [51].

Specifically, immunosuppressive compounds confirmed in secondary screening including cyclosporin A, resatorvid and rapamycin, which have been shown to affect microglial activation by inhibiting calcineurin [52], Toll-like receptor 4 (TLR4) [53] and mechanistic target of rapamycin (mTOR) [54], respectively, and reducing pro-inflammatory cytokine production and neuroinflammation. Pathway enrichment analysis of transcriptomic data indicated that compound treatment strongly suppressed Fc gamma-receptor mediated phagocytosis gene pathways while inducing, to diverse extents, cell activation pathways. This diversity enabled us to select compounds with strong phagocytosis reduction and minimal cell activation.

Similarly, we show that epigenetic modulators, such as the histone deacetylase (HDAC) inhibitor vorinostat, greatly reduce synaptosome phagocytosis and hyper-ramify the piMGLCs with concomitant suppression of gene pathways, e.g., phagosome formation, VEGF signaling, regulation of actin motility, and oxidative phosphorylation. Identified kinase inhibitors, including lapatinib, lazertinib, palbociclib, and dasatinib, target cell signaling pathways that may regulate microglial function and synaptic plasticity.

We further show that antipsychotics identified in our screen, including loxapine and perphenazine, which exert their primary antipsychotic effect through neuronal dopamine receptor antagonism, also diminish microglial phagocytosis function. Other identified neurotransmitter modulators, such as vilazodone, affect the levels of serotonin. While less studied than in their neuronal counterparts, microglia do express serotonergic and dopaminergic receptors and respond to serotonin (HT) and dopamine [55, 56]. Notably, loss-of-function in the 5-HTR_2B_ serotonergic receptor, the predominant HT receptor expressed in microglia, reduced presynaptic material phagocytosis in an engineered mouse model [57]. Our results suggest that these drugs may exert more direct effects on microglia via these pathways, beyond their known neuronal effects. Whether these effects contribute to antipsychotic or mood stabilizing effects in vivo merits further study.

Finally, we identified compounds targeting the mTOR pathway, including rapamycin (sirolimus) and its analog, everolimus, as modulating both synaptic phagocytosis and morphology. These compounds have been shown to reduce microglial pro-inflammatory cytokine production and shift polarization towards an anti-inflammatory state [58, 59], which could reduce synaptic engulfment and neuroinflammation in conditions such as AD [60], schizophrenia [61], and bipolar disorder [62].

Several of the identified compounds have been investigated pre-clinically for neurological conditions, including psychiatric and neurodegenerative disorders, as well as traumatic brain injury (TBI). Examples include SRX246, a potent and highly selective vasopressin 1a receptor antagonist, studied for its potential effects on stress response and mitigation of anxiety and aggression in Huntington’s Disease (HD) patients [63]; SRT2104, a Silent information regulator 1 (SIRT1) activator that promotes cellular survival pathways, reduces oxidative stress, and may slow neurodegenerative progression in disorders including AD and Parkinson’s [64]; and resatorvid (also known as TAK-242), a TLR4 inhibitor, that has been explored in animal models for its ability to reduce neuroinflammation and protect against brain injury by decreasing microglial activation [65], as well as clinical studies of severe sepsis where it was shown to be safe and tolerable, though ineffective for this indication [66].

On the other hand, many of our small-molecule screening hits have not previously been investigated clinically for CNS indications. For example, the tyrosine kinase inhibitors lapatinib, alectinib, and lazertinib have been extensively investigated, but exclusively in the treatment of various cancers [67]. Another example is the HDAC inhibitor vorinostat, which has a favorable safety and tolerability profile when used in monotherapy or in conjunction with other therapies for the treatment of cutaneous T-cell lymphoma (CTCL) [68].

While some of these drugs, including vorinostat [69], have shown promising results in animal models by modulating various microglial functions and potentially impacting neurological pathology, they have not been studied in patients for psychiatric and neurodevelopmental disorders. Pharmacological interventions targeting microglial functions, such as synaptic pruning and activation, warrant further research for their potential to treat symptomatic patients, slow disease progression, or delay and even prevent onset in high-risk individuals. Randomized controlled trials would be needed to establish the clinical utility and safety profiles of these drugs in treating these disorders.

In aggregate, our results identify a range of CNS-penetrant compounds with established human safety profiles. Considering the link between microglial dysregulation and multiple neurodevelopmental, neurodegenerative, and psychiatric disorders [21, 70, 71], these therefore represent high-priority candidates for further study in vivo. More broadly, understanding the precise mechanisms by which these drugs modulate microglial function could open new therapeutic avenues for treating a range of brain diseases. Notably, given their potential to shift pruning trajectories, they may find application not solely in controlling symptoms, but potentially in modifying disease course.

## Supplementary information

Supplementary Materials and Methods

Supplementary Data Table S1

Supplementary Data Table S2

Supplementary Data Table S3

Supplementary Data Table S4

Supplementary Data Table S5

Supplementary Data Table S6

Supplementary Data Table S7

Supplementary Data Table S8

## Data Availability

Gene expression data will be made available for download from the NCBI Gene Expression Omnibus (https://www.ncbi.nlm.nih.gov/geo) upon publication. Additional data that support the findings of this study will be available from the corresponding authors upon reasonable request.
