# Supplementary material for: Identifying brain-penetrant small-molecule modulators of human microglia using a cellular model of synaptic pruning
Source: Neuropsychopharmacology. 2025 May 9;50(10):1544–52. doi: 10.1038/s41386-025-02123-1 (PMC12339681; doi:10.1038/s41386-025-02123-1)
Supplement: Supplementary file 1 — Supplementary Materials and Methods [file 41386_2025_2123_MOESM1_ESM.pdf]

Running Title: Small molecule modulators of human microglia

Supplemental Materials and Methods

Description of Additional Supplemental Data Tables

Supplemental Data Table 1. CNS-penetrant small molecule library of 489 compounds used in primary screen related to Figure 2A.

Supplemental Data Table 2. CNS-penetrant small molecule library stocks and final dilution information related to Figs. 2A and 2B and transcriptomic analyses.

Supplemental Data Table 3. Normalized mean phagocytic index and Z-score (compared to DMSO control) for primary screen as related to Fig. 2A.

Supplemental Data Table 4. Secondary confirmation screen results including phagocytic index (normalized to DMSO control) and measured morphometric parameters: mean eccentricity, solidity and IBA1 intensity related to Figs. 2B, 3B, 3C, 3D, S2 and S3.

Supplemental Data Table 5. Clinical phase status of confirmed compounds resulting in <50% phagocytic index reduction related to Fig. 2B.

Supplemental Data Table 6. Full list of Ingenuity Pathway Analysis (IPA) analysis implicated pathways related to Figs. 5 and S4.

Supplemental Data Table 7. CellProfiler settings used as related to Figs. 1, 2 and 3, Figs. S2 and S3 and Methods.

Supplemental Data Table 8. Multiplexed Drug-seq barcodes used for transcriptomic analysis related to Figs. 4 and 5 and Methods.

Supplemental Figures and Tables:

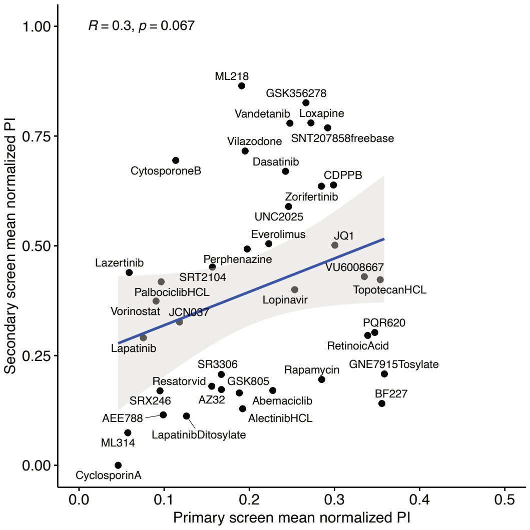

Figure S1. Correlation of measured phagocytic indices for the 40 non-toxic, phagocytosis reducing compounds between the primary and secondary screens.

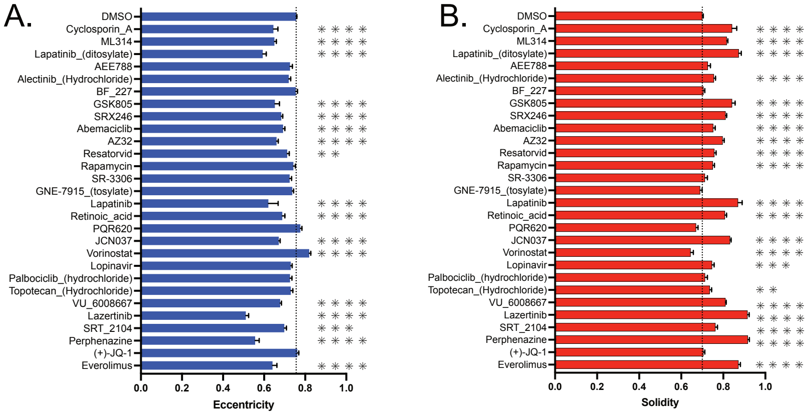

Figure S2. Bars represent the means of image-level piMGLCs (a) eccentricity and (b) solidity values for the 28 compounds confirmed to decrease phagocytosis by 50% or more in the secondary screen. The vertical dotted line represents the DMSO mean value, asterisks signify the adjusted p-values of the dunnett's multiple comparison test, which tests each compound's value to the DMSO control. One to four asterisks correlate to an adjusted p-value of <0.05, <0.01, <0.001, and <0.0001, respectively. Error bars indicate SEM.

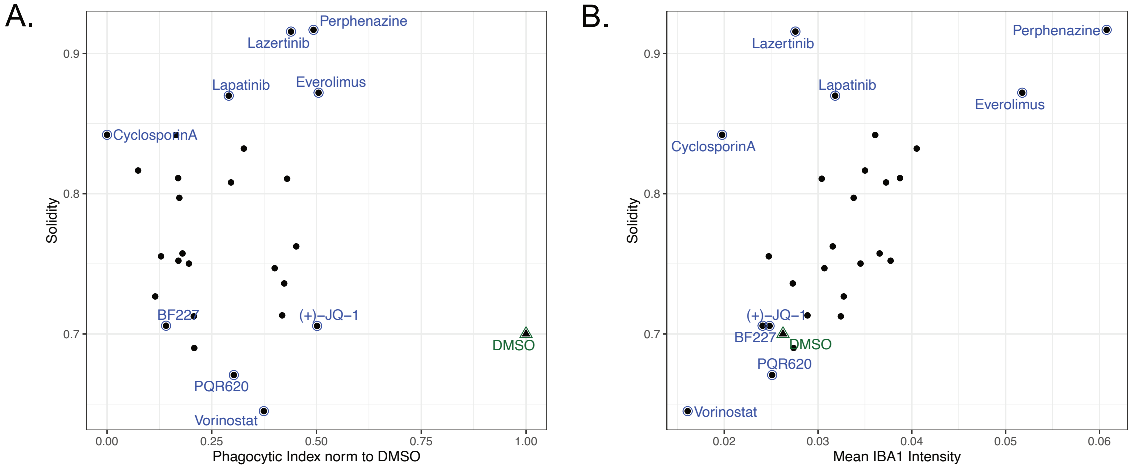

Figure S3. Phenotypic screening identifies compounds that modulate piMGLC morphology. (a) Phagocytic index versus solidity for compounds ≤ 50% DMSO control phagocytic index in secondary screen. (b) IBA1 intensity versus eccentricity for compounds ≤ 50% DMSO control phagocytic index in secondary screen. Each dot in (a) and (b) is mean value of 15 image fields with indicated compounds corresponding to images in Figure 4A for representation.

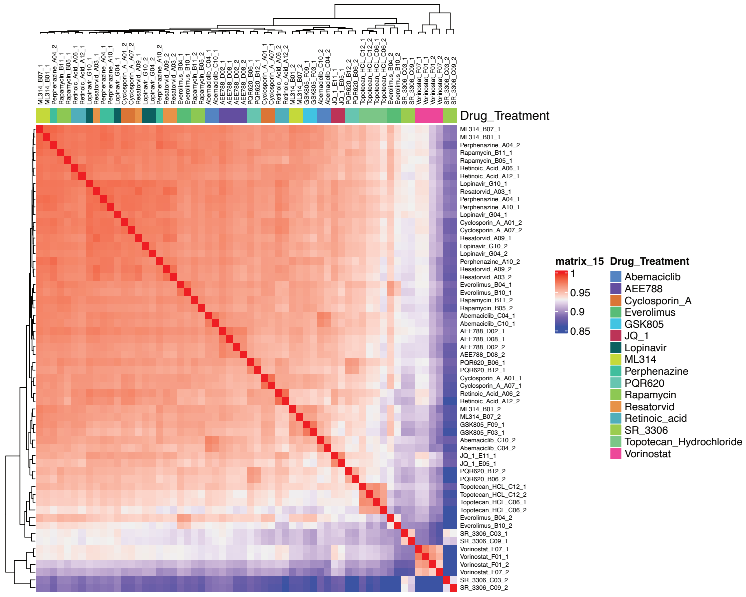

Figure S4. Hierarchical clustering of the Pearson correlation coefficients of all replicates of the 16 RNA-active compounds identified in this study.

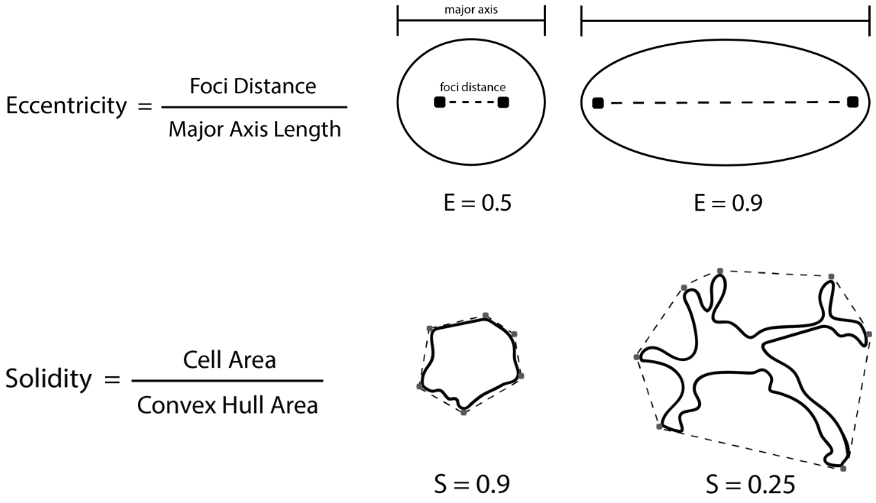

Figure S5. Representative examples of eccentricity and solidity measurements.

Antibody	Species	Vendor (cat#)	Dilution
IBA1	Chicken monoclonal	Synaptic Systems (234009)	1:1000
PU.1	Rabbit monoclonal	Abcam (ab183327)	1:1000
CX3CR1	Mouse polyclonal	Abnova (H00001524-B01P)	1:100
P2RY12	Rabbit polyclonal	Alamone (APR-020)	1:100
Chicken secondary	Goat-Alexafluor647	Invitrogen (A21449)	1:500
Rabbit secondary	Donkey-Alexafluor488	Invitrogen (A21206)	1:500
Mouse secondary	Donkey-Alexafluor488	Invitrogen (A21202)	1:500

Table S1. Antibodies used in this study

Supplemental Methods:

Large-scale isolation of PBMCs from leukapheresis

Peripheral blood mononuclear cells (PBMCs) were isolated from a half leukapheresis pack (leukopak) sourced from HemaCare Corporation (now part of Charles River Laboratories Cell Solutions) using a standard operating procedure from the AIDS Clinical Trials Group Laboratory Technologist Committee (https://www.hanc.info/content/dam/hanc/documents/laboratory/actg-impaact-laboratory-manual/Leukopak%20PBMC%20Processing%20Standard%20Operating%20Procedure%20v2.0_22Nov2022.pdf).

Contents of the leukopak were diluted with Hank's Balanced Salt Solution without Calcium, Magnesium, phenol red (HBSS, Thermo Fisher #14175095) using at least a 1:2 ratio of leukopak to dilutant. 50ml conical tubes were prepared by adding 15ml Density Gradient Medium (DGM, Stemcell Technologies Lymphoprep 07851) to the bottom of each tube. The full volume of the diluted leukopak was split evenly into the 50ml conical tubes, layering the solution on top of the DGM in each tube. Tubes were centrifuged at 400g for 30 minutes, with centrifuge brake set to "off". The PBMC fractions between the plasma and DGM were transferred to new tubes and diluted with HBSS to a final volume of 45ml each. A series of washing steps followed, starting with a centrifugation at 300g for 10 minutes, again with no brake. The supernatants were discarded, and pellets resuspended in 5ml HBSS, before combining into 2 x 50ml conical tubes. This wash was repeated, and cells were consolidated into a single conical tube with a 20ml volume. Cell concentration was quantified using a Countess II automated cell counter (ThermoFisher), and the volume of Cryostor CS10 medium (Sigma-Aldrich C2874) was calculated to aliquot PBMC's at

25 million cells/ml. The final centrifugation at 300g for 10 minutes was completed and the pellet was resuspended in the Cryostor cs10 medium, before slow freezing in a Mr. Frosty (ThermoFisher #5100-0001) freezing container at -80C and moving to long term liquid Nitrogen storage the following day. The cells were suspended in Cryostor cs10 at room temperature for a maximum of 10 minutes before being added to the freezer. If the time to aliquot this suspension was estimated to be longer, the cell solution was split into multiple batches before the final centrifugation, and aliquots were prepared one batch at a time.

PBMC-derived induced microglia-like cell (piMGLC) batch culture and assay plate seeding

Frozen and aliquoted peripheral blood mononuclear cells (PBMCs) isolated as described above from a healthy control donated half leukopak (Charles River, Lowell MA / Hemacare, Northridge CA) were quick-thawed in a 37C water bath and immediately transferred into RPMI-1640 (Sigma, #R8758) + 10% heat-inactivated fetal bovine serum (Sigma, #12306C) + 1% of Penicillin/Streptomycin (Life Technologies, cat# 15140-122). Cells were washed by centrifugation at 300 g for 5 min with the brake off and resuspended in fresh media, then counted and plated at a density of approximately 400,000 cells/cm^2 in a tissue culture treated 6-well plate (Corning, #353046) pre-coated with Geltrex (Gibco, #A1413202) for 1 hour. After incubating for 24 hours, the media was carefully removed and replaced with RPMI-1640 + 1% Penicillin/Streptomycin + 1% Glutamax (Life Technologies, # 35050-061) + 100 ng/ml IL-34 (Biolegend Inc, #577904) + 10 ng/ml GM-CSF (PeproTech, #300-03). After a 10-day incubation period for trans-differentiation, culture media was collected and filtered with a 0.22um Steriflip-GP Filter (EMD Millpore #SCGP00525) and retained. PBMC-derived induced microglia-like cells (piMGLCs) were harvested using Accutase (Sigma, #A6964) for 5 minutes at 37C to detach. They

were then centrifuged at 300g for 5 min and counted so they could be resuspended in the proper amount of retained and filtered cell culture media to 50,000 cells/ml, and plated into 96-well tissue culture plates (Corning, #3904) at a density of 30,000 cells/cm^2 (10,000 cells per well in 200ul) for 4 days before assaying. If the assay plates required a higher volume of cell-culture media than that collected from the batch of 6-well plates, supplementary fresh media of RPMI-1640 with Penicillin/Streptomycin and Glutamax would be used to reach the required seeding volume.

Large scale generation of synaptosomes from iPSC-derived neural cultures for use in models of synaptic pruning screening assays.

iPSCs were reprogrammed from fibroblasts and used to derive neural progenitor cells (NPCs), which were differentiated into neural cultures, as previously described.(1-4) Human iPSC-derived neural progenitor cells (NPC's) cultured in neural expansion media (50% Advanced DMEM/F12, 50% Neurobasal, and neural induction supplement – Gibco: 21103-049, 12634-010, A16477-01) were expanded and seeded (50 million per flask) into T1000 flasks (Millipore, #PFHYS1008) for large-scale neuronal differentiation. NPCs were differentiated in neuronal differentiation medium (Neurobasal media (Gibco # 21103049) supplemented with 1× each (N2 supplement (Stemcell Technologies SCT # 7156), B27 supplement without Vitamin A (Gibco # 12587010), non-essential amino acids (NEAA Gibco # 11140050), penn/strep, 1 μM ascorbic acid, 10 ng/mL BDNF and GDNF (Peprotech), and 1 μg/mL mouse laminin (Sigma # L2020) replacing media weekly for 8-weeks. 24 hours before isolation, media was replaced with human Astrocyte-conditioned medium (ScienceCell, #1811). To start the isolation, media was aspirated and replaced with 1X gradient buffer (0.32M sucrose, 0.75mM $NaHCO_3$, 5mM Tris in Milli-Q water) + HALT protease and phosphatase inhibitor cocktail (Thermo Scientific, #78440), before shaking the flasks

to detach the neural cultures from the surface layers of the flask. Cell suspensions were transferred to a dounce homogenizer (Bellco Glass, #1984-10015) and homogenized using 12 up and down strokes per 10ml using the tight plunger. Homogenate was split between 15ml conical tubes and centrifuged at 700g, 4°C, for 10 minutes. Supernatants were saved and the pellets were resuspended in 1X gradient buffer to repeat the previous step once more. Supernatants were combined and transferred to high-speed centrifuge tubes (Beckman Coulter, #344058) to spin at 15000g, 4°C, for 15 minutes. Sucrose gradients were prepared in ultracentrifuge tubes by layering 0.85M sucrose on top of 1.28M sucrose. After centrifugation, the pellets were resuspended in 1X gradient buffer and carefully added to the ultracentrifuge tubes as the top layer of the sucrose gradients. Gradients were centrifuged at 26500 rpm in an ultracentrifuge (Beckman Coulter optima L-90K) using a pre-chilled rotor and swinging cups (SW 32 Ti) for 2 hours at 4°C, with no brake. To collect the synaptosome fraction between the 1.28M and 0.85M sucrose layers, a 5ml syringe with 16G needle was used to pierce the wall of the tube at the level of the synaptosome band to aspirate the fraction, taking as little surrounding gradient buffer as possible. The collected fractions were diluted (at least 1:5) with 1mM $NaHCO_3$ Milli-Q water and centrifuged a final time at 20000g, 4°C, for 20 minutes. Final pellets were resuspended in 1X gradient buffer + 1mg/ml BSA, and aliquoted into cryovials at 100μl/tube. Protein concentration of isolated synaptosomes was quantified using a Pierce BCA protein assay kit (Thermo Scientific, #23225).

Compound treatments

24 hours before the start of the assay, the media volume per well was brought to 150ul and piMGLCs were pretreated with the 489 screening library compounds (CNS-penetrant compound library, MedChemExpress #HY-LO28) (Supplemental Data Table S1) at a final concentration of

10μM, with some exceptions at 2μM (Supplemental Data Table S2), by diluting the compounds in basal RPMI-1640 and adding 50ul of one diluted compound per well. For the primary screen a single dilution per compound was used in a single well per plate, with three replicates split across different plates. DMSO (0.1%) was used as a vehicle control and was used in 3 replicate wells on each plate. The secondary screen included piMGLC plates treated with 47 compounds for 24-hours (Supplemental Data Table S4).

Phagocytosis assays

Phagocytosis assays were performed in 96-well plates (Corning, #3904) containing piMGLCs at a density of 30,000 cells/cm^2 (10,000 cells per well in 200ul). Human synaptosomes were thawed at room temperature and an equal volume of 0.1M sodium bicarbonate, pH 9 was added to the synaptosomes. pHrodo-Red (Invitrogen, #P36600, 6.67ug/ul) was added at a protein ug ratio of 1:2 (dye:synaptosome). The labeling reaction was incubated at room temperature for 1 hour in the dark. Synaptosomes were washed by adding at least an equal volume of PBS, pH 7.4 to the tube and pelleting at 12,000 rpm for 15 min. The wash solution was discarded and the labeled synaptosomes were resuspended in a volume of basal RPMI-1640 to a final concentration of 0.15 μg synaptosomes per μl. Labeled synaptosomes were sonicated in a Branson 1800 (Emerson, #M1800) at 40 kHz for 1 hour. During this time, some wells of microglia were treated with cytochalasin-D (Sigma, #C2618) to a final concentration of 2 μM as a control treatment to inhibit phagocytosis. Following sonication, the synaptosomes were added to wells at a final concentration of 3μg/well. Due to the scale of the primary screen, synaptosomes were added to plate wells using a Multidrop Combi reagent dispenser for this experiment (Thermo Scientific, 5840340). Synaptosome were added to wells using a multichannel pipette for experiments following the

primary screen. Phagocytosis assays were ended after 3 hours by fixation with 4% paraformaldehyde (Electron Microscopy Sciences, #15713S).

High content image analysis

Fifteen randomly spaced confocal microscopy images per well were analyzed using CellProfiler (Version 4.2.1).(5) Specific pipeline parameters used are detailed in Supplemental Data Table S7. CellProfiler pipeline files used will be made available at https://cellprofiler.org/published-pipelines upon publication. Briefly, illumination correction was used to decrease background signal in the images, nuclei and piMGLC structures were segmented and masked to each other, synaptosomes were segmented and masked to the cytoplasm, and all descriptive calculations such as signal intensity and object size for each of the identified objects were exported. The pipeline was fine-tuned to ensure optimal object segmentation accuracy, and steps were added to the downstream analysis to handle any object segmentation errors that did occur. The same pipeline was used for the analysis of all experiments, and only the lower bounds of the automated Otsu thresholding for synaptosome segmentation were adjusted between experiments since each may have a different background fluorescent signal intensity. To set the optimal lower bound on the thresholding and prevent background fluorescence from being included in synaptosome segmentation, 2uM cytochalasin D treated wells (phagocytosis inhibitor used as positive control) were included in each plate to have a low signal well to calibrate the thresholding bounds.

Field level filters were applied for identified cell number and PI, next fields were aggregated to wells and similar well-level filters were applied. Data exported from CellProfiler were loaded into an R notebook for cleaning. Initially, cell number was used to identify toxic treatments. Further, we identified potential CellProfiler segmentation errors. Next, a filter was applied to retain only

images with between 4 and 80 cells, thus omitting the extremely dense or sparse fields that likely result from CellProfiler over-segmentation or treatment toxicity, respectively. The next filter removed image fields where the phagocytic index (synaptosome area divided by cell count) was greater than the mean plus three times the standard deviation (3SD), which would be a result of inaccurate synaptosome segmentation.

The secondary confirmatory compound screen used the image derived data at this more sensitive image field granularity, with a minimum of 5 image fields remaining after the cleaning steps noted above. The primary screen used well-level quantification to reduce noise in the larger scale experiment. To do this the well-level phagocytic index was calculated as the sum of synaptosome area divided by the sum of cells in all images per well. Similar cleaning filters were applied to the well-level data. First, the same max phagocytic index threshold of mean plus 3SD was used. Along with this, wells with low per-well cell counts were omitted using a minimum count threshold of 10% of the mean of cell counts in DMSO treated wells. Compounds with fewer than three remaining replicate wells after applying these filters were omitted from further analysis.

Multiplexed library preparation and RNA sequencing

96-well multiplexed libraries (DRUG-seq (6)) were prepared using the MERCURIUS™ DRUG-seq kit (Alithea Genomics, 10841) following manufacturer instructions. Briefly, cells were washed using 100µL per well of Dulbecco's Phosphate Buffered Saline (Gibco, 14190-144). Cells were then lysed for 10-15 minutes in 20µL per well of prepared and chilled 1x Cell Lysis Buffer at 4°C. Lysate was carefully removed from the cell culture plate and transferred to a 96-well PCR plate, then spun at 300xg for 5 min in a centrifuge pre-chilled to 4°C. Up to 20µL of supernatant was then removed to a clean 96-well PCR plate for storage at -80°C for up to one month.

For reverse transcription, stored lysate was thawed at 4°C, then 10µL of lysate from each well was transferred to a 96-well plate containing dry, well position specific barcoded oligo-dT primers (Alithea Genomics, 10513). Upon resuspension of the oligo-dT primers, 10µL of prepared RT Master Mix was added to each well and reverse transcription was carried according to provided instructions (incubation for 30min at 50°C, 10min at 85°C, then cooling to 4°C). Resulting first-strand cDNA was collected and pooled, mixed with 7x volume of DNA binding buffer (Zymo, D4003-1-L), and purified on a Zymo-Spin IC column (Zymo, C1004-50). Purified first-strand cDNA was eluted in 20µL nuclease-free water. Excess oligo-dT was digested from 17µL of eluate by adding Exonuclease I Enzyme and Buffer and incubating for 30min at 37°C, 20 min at 80°C, then cooling to 4°C. Second-strand synthesis was carried out immediately after by adding 7µL of prepared Second Strand Synthesis reaction mix and incubating for 20 min at 37°C, 30 min at 65°C, and then cooling to 4°C.

Resulting second-strand cDNA was purified using CleanNGS DNA & RNA Clean-Up Magnetic Beads (Bulldog Bio, CNGS005) at a ratio of 0.6x bead slurry to cDNA. Bead purification was carried out using a 0.2 mL PCR Strip Magnetic Separator (Permagen, MSR812). Upon purification, cDNA was measured using Qubit 1X dsDNA HS Assay kit (Invitrogen, Q33231) and 50-60ng of cDNA was tagmented in a 20µL reaction with 4µL of provided Tagmentation Enzyme and 4µL of Tagmentation Buffer. After incubating at 55°C for 7 minutes, the tagmented library was brought to 50µL and purified with CleanNGS beads at a ratio of 0.6x bead slurry to cDNA. Finally, the tagmented library was pre-amplified with provided UDI adapters included in kit for 10 cycles (one cycle composed of 10 seconds at 98°C, 30 seconds at 63°C, and 1 min at 72°C) total. In preparation for sequencing, this pre-amplified library was purified twice with CleanNGS beads at a ratio of 0.7x bead slurry to cDNA and eluted in 20uL.

Quality control and measurement of library concentration of finished libraries was performed with Agilent 5300 Fragment Analyzer System using the HS NGS Fragment Kit (Agilent, DNF-474-0500), for quality control and measurement of library concentration. Library was loaded onto one lane of a NovaSeq X Plus 1.5B read flow cell and sequenced on the NovaSeq X Plus instrument. Sequencing cycle structure was as recommended by manufacturer (Read 1: 28 cycles; i7 read: 8 cycles; i5 read: 8 cycles; Read 2: 90 cycles). Samples were split over two different sequencing runs, with at least one replicate of each studied compound on each run. Sequencing resulted in 615 million read pairs for run one and 1016 million read pairs for run two.

DRUG-seq data analysis

Sample demultiplexing and read alignment was done following the data analysis pipeline steps in MERCURIUS DRUG-Seq kit documentation. Briefly, raw reads were aligned to reference genome hg38 using STAR v. 2.7.11b (7) using "solo" mode. Unique molecular identifier count matrices were generated in this step by supplying the list of sequencing barcodes to option –soloCBwhitelist (Supplemental Data Table S8). The resulting demultiplexed count matrix contained on average 3 million $\pm$ 1 million reads per sample and was used for downstream analysis.

Differential Expression analysis was done using a modified version of the DRUG-seq analysis pipeline (8) using DESeq2 (9) to normalize the count matrix, fit a negative binomial regression model with design = ~ batch + compound, and perform differential expression analysis using the Wald test. Genes were considered significantly differentially expressed with adjusted p-value < 0.1 and absolute log2 fold-change > 1, using each compound and DMSO as comparison groups. Briefly, using all DMSO wells, we determine the "best" DMSO wells by performing 500 random

DMSO vs. DMSO comparison, and selecting the two DMSO replicates from each group that contributed to the fewest differentially expressed genes (DEG). Next, we determine the null distribution of DEG by comparing 500 randomly sampled sets of DMSO replicates to our chosen 4 "best" DMSO wells. Finally, we compare all replicates of each treatment compound vs. the 4 best DMSO wells. Treatment compounds were considered "RNA active" if the number of DEG resulting from the comparison with DMSO was greater than the 95% of DMSO vs. DMSO comparisons, which was determined to be 179 DEG. Uniform Manifold Approximation and Projection (UMAP) dimension reduction was used to visualize overall similarity of transcriptional profile of all transcriptionally active treatment wells. Functional enrichment analysis for all sets of treatment compound vs. DMSO comparisons DEGs was performed using QIAGEN Ingenuity Pathway Analysis v. 24.0.1 (10) canonical pathway analysis. Original code developed for this study have been deposited in the public repository https://github.com/rbatorsky/drugseq_perlis.

Immunofluorescence

Live cells were fixed with 4% paraformaldehyde for 15 min at room temperature. Cells in a 96-well format were washed with 100 ul of Wash Buffer, PBS + 0.5% FBS, three times. Then 100 ul of Block and Permeabilization Buffer, PBS + 0.5% FBS + 0.3% Triton-X, were added per well. Wells were incubated at room temperature for 1 hr, then washed three times with Wash Buffer. Primary antibodies were diluted in Antibody buffer, PBS + 0.5% FBS + 0.1% Triton-X, added to the wells and incubated for 1 hr at room temperature or overnight at 4 C. Wells were washed three times with Wash Buffer. Secondary antibodies were diluted in Antibody Buffer and added to wells and incubated for 45 min at 4 C. After a final three washes, the wells were imaged with an IN Cell

Analyzer 6000 automated confocal microscope (Cytiva) at 20X. Details of antibodies used in Table S1.

Morphometric Image Analysis

Measurements used to characterize cell morphology included eccentricity and solidity. Eccentricity is calculated using an ellipse encompassing the cell and taking the ratio of the distance between ellipse foci and the length of its major axis. This results in a value between 0-1 where long, flatter cells are close to 1, and circular cells are closer to 0.5 (cell morphology never approaches a perfect circle to have a value close to 0).

Solidity is calculated using the object's convex hull, the smallest possible convex shape that contains the entire object. Solidity is the ratio of the cell's area to the area of its convex hull, which means more circular cells have a solidity close to 1, while ramified cells with many branches result in solidity values closer to 0. Figure S5 shows representative examples of these measurements.

Supplemental References:

1. Sellgren CM, Gracias J, Watmuff B, Biag JD, Thanos JM, Whittredge PB, et al. Increased synapse elimination by microglia in schizophrenia patient-derived models of synaptic pruning. Nat Neurosci. 2019;22(3):374-85. doi:10.1038/s41593-018-0334-7
2. Sheridan SD, Horng JE, Yeh H, McCrea L, Wang J, Fu T, et al. Loss of function in the neurodevelopmental disease and schizophrenia-associated gene CYFIP1 in human microglia-like cells supports a functional role in synaptic engulfment. Biological Psychiatry. 2024;95(7):676-86.
3. Sheridan SD, Thanos JM, De Guzman RM, McCrea LT, Horng JE, Fu T, et al. Umbilical cord blood-derived microglia-like cells to model COVID-19 exposure. Translational psychiatry. 2021;11(1):1-9.
4. Sellgren C, Sheridan S, Gracias J, Xuan D, Fu T, Perlis R. Patient-specific models of microglia-mediated engulfment of synapses and neural progenitors. Molecular Psychiatry. 2017;22(170-177).
5. Stirling DR, Swain-Bowden MJ, Lucas AM, Carpenter AE, Cimini BA, Goodman A. CellProfiler 4: improvements in speed, utility and usability. BMC Bioinformatics. 2021;22(1):433. doi:10.1186/s12859-021-04344-9

6. Ye C, Ho DJ, Neri M, Yang C, Kulkarni T, Randhawa R, et al. DRUG-seq for miniaturized high-throughput transcriptome profiling in drug discovery. Nat Commun. 2018;9(1):4307. doi:10.1038/s41467-018-06500-x
7. Dobin A, Davis CA, Schlesinger F, Drenkow J, Zaleski C, Jha S, et al. STAR: ultrafast universal RNA-seq aligner. Bioinformatics. 2013;29(1):15-21.
8. Li J, Ho DJ, Henault M, Yang C, Neri M, Ge R, et al. DRUG-seq Provides Unbiased Biological Activity Readouts for Neuroscience Drug Discovery. ACS Chemical Biology. 2022;17(6):1401-14. doi:10.1021/acschembio.1c00920
9. Love MI, Huber W, Anders S. Moderated estimation of fold change and dispersion for RNA-seq data with DESeq2. Genome biology. 2014;15:1-21.
10. Krämer A, Green J, Pollard Jr J, Tugendreich S. Causal analysis approaches in ingenuity pathway analysis. Bioinformatics. 2014;30(4):523-30.
