# Supplementary material for: Identifying brain-penetrant small-molecule modulators of human microglia using a cellular model of synaptic pruning
Source: Neuropsychopharmacology. 2025 May 9;50(10):1544–52. doi: 10.1038/s41386-025-02123-1 (PMC12339681; doi:10.1038/s41386-025-02123-1)
Supplement: Supplementary file 3 — Supplementary Data Table S2 [file 41386_2025_2123_MOESM3_ESM.pdf]

Table S2. Other Drug Treatment Information
All compounds dissolved in DMSO and used at 10uM except for the following:

Compound Name	Solvent	Concentration
**Primary Screen**		
**Hoechst 34580 (tetrahydrochloride)**	DMSO	2uM
**S 38093**	DMSO	2uM
**Gatifloxacin**	DMSO	2uM
**Droxidopa**	DMSO	2uM
**Itraconazole**	DMSO	2uM
**SRT 2104**	DMSO	2uM
**Tenofovir**	DMSO	2uM
**Baclofen**	DMSO	2uM
**Abemaciclib**	DMSO	2uM
**Palbociclib (hydrochloride)**	Water	10uM
**Fosphenytoin (disodium)**	Water	10uM
**Gabapentin**	Water	10uM
**Secondary Screen**		
**SRT_2104**	DMSO	2uM
**Abemaciclib**	DMSO	2uM
**Palbociclib_(hydrochloride)**	Water	10uM
**Drug-Seq**		
**Abemaciclib**	DMSO	2uM
